# An *in vitro *study comparing a peripherally inserted central catheter to a conventional central venous catheter: no difference in static and dynamic pressure transmission

**DOI:** 10.1186/1471-2253-10-18

**Published:** 2010-10-12

**Authors:** Heath E Latham, Timothy T Dwyer, Bethene L Gregg, Steven Q Simpson

**Affiliations:** 1 Division of Pulmonary and Critical Care Medicine, Department of Medicine, University of Kansas Medical Center, Kansas City, Kansas, USA; 2 Division of Respiratory Care Education, University of Kansas Medical Center, Kansas City, Kansas, USA; 3 3901 Rainbow Blvd, MS 3007, Kansas City, KS 66160, USA

## Abstract

**Background:**

Early goal directed therapy improves survival in patients with septic shock. Central venous pressure (CVP) monitoring is essential to guide adequate resuscitation. Use of peripherally inserted central catheters (PICC) is increasing, but little data exists comparing a PICC to a conventional CVP catheter. We studied the accuracy of a novel PICC to transmit static and dynamic pressures *in vitro*.

**Methods:**

We designed a device to generate controlled pressures via a column of water allowing simultaneous measurements from a PICC and a standard triple lumen catheter. Digital transducers were used to obtain all pressure readings. Measurements of static pressures over a physiologic range were recorded using 5Fr and 6Fr dual lumen PICCs. Additionally, random repetitive pressure pulses were applied to the column of water to simulate physiologic intravascular pressure variations. The resultant PICC and control waveforms were recorded simultaneously.

**Results:**

Six-hundred thirty measurements were made using the 5 Fr and 6 Fr PICCs. The average bias determined by Bland-Altman plot was 0.043 mmHg for 5 Fr PICC and 0.023 mmHg for 6 Fr PICC with a difference range of 1.0 to -1.0. The correlation coefficient for both catheters was 1.0 (p-value < 0.001). Dynamic pressure waveforms plotted simultaneously between PICC and control revealed equal peaks and troughs.

**Conclusion:**

*In vitro*, no static or dynamic pressure differences were found between the PICC and a conventional CVP catheter. Clinical studies are required to assess whether the novel PICC has bedside equivalence to conventional catheters when measuring central venous pressures.

## Background

Sepsis is a major cause of death in the world and carries a mortality rate of 20 to 60% depending on the severity of the disease [1]. Severe sepsis and septic shock are the leading cause of death in non-cardiac intensive care units and the 10^th ^overall cause of death in the United States [2]. As the country's population grows and ages, the incidence of sepsis is also increasing [1-3]. Despite profound technological advancements in medicine over the last two decades, no intervention has impacted the treatment of severe sepsis and septic shock to the degree of early goal directed therapy. In 2001, Rivers and colleagues demonstrated a sixteen percent reduction in hospital mortality with an early intervention bundle including aggressive volume resuscitation guided by central venous pressure (CVP) monitoring [4].

Centrally inserted central catheters (CICC) and pulmonary artery catheters (PAC) are the current gold standard instruments for measuring CVP, but insertion of these catheters carries the risk of pneumothorax, hemothorax, and severe bleeding [5]. Peripherally inserted central catheters (PICC) are increasingly used in the hospital setting, and do not have the same risk of complications with insertion as compared to centrally inserted catheters [6]. Interestingly, CVP monitoring is an indication for use for several commercially available PICCs, including those manufactured by AngioDynamics, Arrow, Bard, and Medcomp [7-11]. However, there is limited literature on functional accuracy of PICCs for measuring CVP [12,13]. In addition, PICC length and flexibility, necessary design requirements, intuitively suggest to clinicians that central venous pressure measurement via PICC may not be accurate.

For our study, we selected the AngioDynamics Morpheus PICC. A property unique to the Morpheus catheter is that the shaft transitions from increased stiffness at the proximal end to softer flexibility at the distal end [14].

## Purpose

The purpose of our study was to assess the accuracy of static and dynamic pressures measured via the Morpheus PICC compared to a conventional catheter for central insertion. We hypothesized that under *in vitro *conditions, pressure transmission through the PICC would be equal to pressure transmission through the conventional catheter.

## Methods

In an *in vitro *study, we designed an inverted T-device to generate controlled pressures via a column of water. The PICC and control were inserted into the device opposite of each other with the catheter tips at the base of the column of water, allowing simultaneous measurements from the PICC and control catheter. Care was taken to avoid excessive external compression or bending of the catheters at the insertion points. The column of water was calibrated and could be automatically adjusted to generate static pressures over a physiologic range of 5 to 25 mmHg. Pressures were measured using TruWave pressure transducers (Edwards Lifesciences LLC. Irvine, CA) and recorded using an Agilent Technologies (Andover, MA) model V24C monitor. The transducers for the PICC and control were mounted level with each other, and the base of the water column. Standard pressure bags and pressure tubing with infusion rates of 3 milliliters per hour were used with each transducer and connected to the catheters via standard luer-lock connections.

We obtained ten 5 Fr and five 6 Fr dual-lumen open ended 65 cm Morpheus PICCs (AngioDynamics, Inc. Queensbury, NY) to be compared to standard Arrow 7 Fr triple lumen catheters (Arrow Multi-Lumen 7 Fr, 20 cm; Teleflex Inc. Reading, PA). The PICC lumens were 18 gauge in diameter and the control lumen was 16 gauge in diameter. The peripherally inserted central catheters were donated by the AngioDynamics Corporation without restriction. Pressures were recorded from each PICC lumen in triplicate at pressures of 5, 8, 10, 12, 15, 20, and 25 mmHg. Static pressure measurements were recorded from the monitor five seconds after the column of water was adjusted to the desired pressure.

Dynamic pressures were also measured. A device capable of applying random repetitive pressure pulses was fitted to the column of water to produce dynamic pressure waveforms up to three hundred cycles per minute. The waveforms were simultaneously recorded at twenty-five millimeters per second on standard grid paper. We compared the 5 Fr and 6 Fr Morpheus PICCs to the central port of an Arrow 7 Fr triple lumen catheter. The waveforms were recorded for a total of ten seconds.

Statistical analysis was performed using a Bland-Altman plot to determine the average bias, standard error, and difference range between measurements obtained via PICC and control catheters. In addition, a correlation coefficient was calculated to determine the strength of relationship between paired data from the PICC and control. A p-value of less than 0.05 was considered significant.

## Results

**Figure 1:**
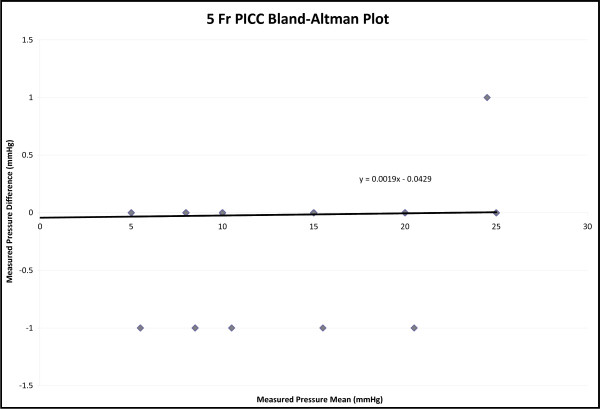
**5 French PICC Bland-Altman Plot**. The average bias determined by the plot is 0.043 mmHg with a standard error of zero. The difference range is -1 to 1 mmHg.

**Figure 2:**
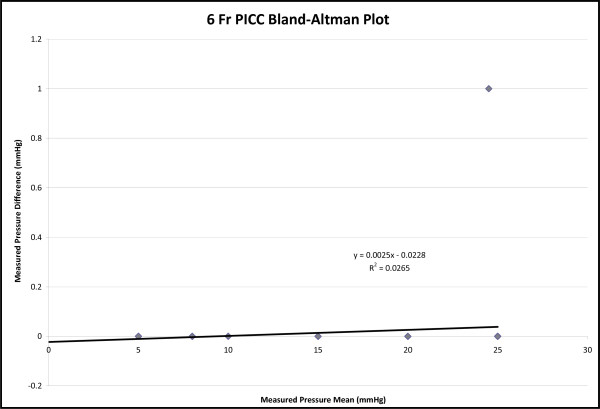
**6 French PICC Bland-Altman Plot**. The average bias determined by the plot is 0.023 mmHg with a standard error of zero. The difference range is 0 to 1 mmHg.

**Figure 3:**
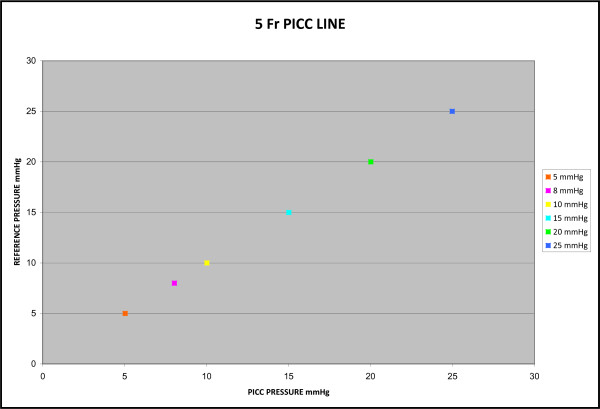
**5 French PICC Static Pressure Scatter Plot**. Four hundred and twenty measurements comparing the pressure recorded from ten 5 Fr PICCs on the x-axis and the control pressure on the y-axis. The correlation coefficient between the two catheters was 1.0 which results in a perfectly linear correlation and a y-intercept of essentially zero.

**Figure 4:**
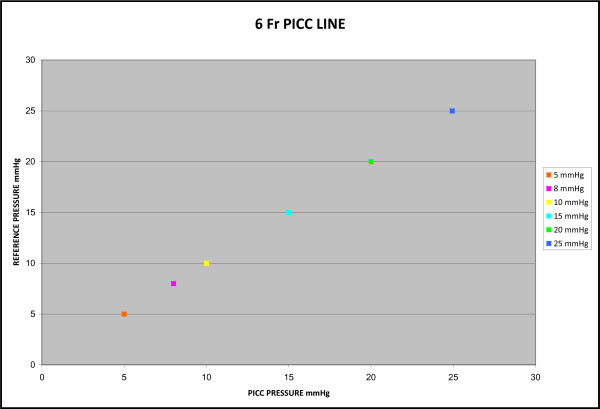
**6 French PICC Static Pressure Scatter Plot**. Two hundred and ten measurements comparing the pressure recorded from five 6 Fr PICCs on the x-axis and the control pressure on the y-axis. The correlation coefficient between the two catheters was 1.0 which results in a perfectly linear correlation and a y-intercept of essentially zero.

A total of six-hundred and thirty measurements were recorded. Four-hundred and twenty measurements were recorded with the 5 Fr PICC and two-hundred and ten using the 6 Fr PICC. The average bias determined by Bland-Altman plot was 0.043 mmHg with a standard error of 0 mmHg for the 5 Fr PICC and 0.023 mmHg with a standard error of 0 mmHg for the 6 Fr PICC. The difference range for both catheters was 1.0 to -1.0 mmHg (Figures 1 and 2). The correlation coefficient of both the 5 Fr and 6 Fr catheters was 1.0 with a p-value < 0.001. The y-intercept was essentially zero when measurements from the two catheters were plotted against each other, with a slope of one (Figures 3 and 4).

**Figure 5:**
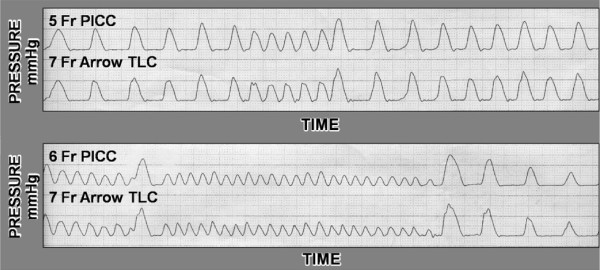
**Pressure Waveforms**. Dynamic pressure waveforms comparing the 5 Fr and 6 Fr PICC to the 7 Fr triple lumen control. There is some phase delay in the PICC waveform. However, the peaks, troughs, and means are equal even at 300 cycles per minute.

There was mild phase delay of the waveform when comparing the PICC to the standard triple lumen catheter during dynamic pressure measurements (Figure 5). However, the peak and trough of the waveforms were equal within the limits of the measurement of the system, and accurately measured changes at three hundred cycles per minute.

## Discussion

Our results confirm our hypothesis that there is no difference between static and dynamic pressures when measured *in vitro *with a PICC as compared to a conventional triple lumen CVP catheter. Both analyses by Bland-Altman plot and the calculated correlation coefficient demonstrated a very high correlation between measurements obtained by the PICC and control catheters. In addition, a standard error of zero and a y-intercept of essentially zero with a slope of 1 confirm agreement between the PICC and control catheters.

Our study contributes to the limited literature on CVP measurement via PICC. Multiple commercially available PICCs carry indications for CVP monitoring, yet Black and colleagues are the only group to demonstrate CVP monitoring equivalency, in clinically stable ICU patients, between a Bard Per-Q-Cath PICC and CICC [7-12]. Similar to Black, we demonstrated *in vitro *that an alternative catheter, the AngioDynamics Morpheus PICC, accurately transmitted static and dynamic pressures across a physiologic range. Further testing is needed to confirm these results in clinical settings, but these findings support the hypothesis that CVP monitoring via PICC is feasible.

The Morpheus PICC is structurally unique, which may contribute to its accuracy. Unlike other models of PICC, the shaft transitions from increased stiffness at the proximal end to softer flexibility at the distal end [14]. The rigidity of the proximal end likely reduces intra-luminal resistance and should endure external compression that could occur in the subcutaneous tissue as the catheter traverses from the insertion site to point of vascular penetration. This rigidity may also prevent compression of the catheter in the region of the subclavian vein which is a known pinch site for vascular catheters. Currently there are no studies comparing different brands of PICC to determine if structural characteristic contribute to accuracy of CVP measurement. However, this could certainly be addressed in future research.

Clinicians are biased against the use of PICCs for hemodynamic monitoring because of their overall length and pliable physical properties. In 2000, Black and colleagues confirmed that both of these properties increase resistance in peripherally inserted catheters [12]. Our study demonstrates that with low flow pressure transducers, the transmission of pressure under both static and dynamic conditions from the tip of the catheter to the transducer is no different with this PICC than with standard central catheters used for pressure monitoring.

Our study does have limitations. It is the first to evaluate the AngioDynamics Morpheus PICC, so no comparison data can be referenced. In addition, our study is limited by its *in vitro *design. Curvature of catheters through the peripheral vasculature and the potential for clot formation on the tip of catheters could not be reproduced in our study. Physicians should use care in considering the potential application of our findings in the clinical setting. Overall there is limited research on hemodynamic monitoring with other brands of peripherally inserted central catheters [12,13]. However, we demonstrated *in vitro *the PICC accurately measures static pressures when compared to standard triple lumen catheters. Even more impressive was the correlation of the dynamic pressure waveforms between the PICC and control. As a result, our study provides a firm background to guide future research in hemodynamic monitoring via PICC in the clinical setting, and if confirmed *in vivo*, our data have implications for the treatment of severe sepsis and other shock states.

The cornerstone of early goal directed therapy in severe sepsis and septic shock is aggressive volume resuscitation guided by serial central venous pressure and Sv02 or ScvO2 measurements [4,15]. Central venous access has been required for measuring both of these parameters and central line placement frequently serves as an obstacle to initiating early goal directed therapy. Multiple barriers to standard central line insertion include difficult landmark identification in morbidly obese patients, impaired pulmonary status unable to tolerate further pulmonary compromise due to pneumothorax, and level of physician comfort with central line placement[5]. Peripherally inserted central catheters may be a means to deliver early goal directed therapy in that a PICC can be placed quickly and without risk of pneumothorax or major bleeding by specially trained nurses[16]. However, PICCs are not risk free. The most common complications associated with PICCs are malposition, line fracture, thrombosis, phlebitis, and catheter related blood stream infection (CRBSI)[17]. Of these, thrombosis and CRBSI are the most concerning. Trerotola and colleagues recently reported a thrombosis rate of 20 percent in critically ill patients, but other studies report rates less than half that [18-20]. In addition, catheter related blood stream infections are not increased with a PICC compared to CICC [19,21-24]. Thus, peripherally inserted catheters appear to be a safe alternative to centrally inserted catheters.

## Conclusion

In conclusion, we believe our study provides documentation of the functional accuracy of PICCs as compared to conventional triple lumen catheters inserted centrally. Peripherally inserted central catheter use in the acute hospital setting is increasing, and complications associated with the insertion procedure of PICCs are less significant compared with standard central lines. If PICCs were determined to be equivalent to CICCs for CVP monitoring *in vivo*, then it is possible that these catheters could be quickly and safely inserted into patients to augment early goal directed therapy. Clearly further studies, especially *in vivo *studies comparing the AngioDynamics Morpheus PICC and other similar catheters to conventional centrally inserted catheters, are needed to confirm their accuracy in patients, especially critically ill patients.

## Abbreviations

CVP: central venous pressure; CICC: centrally inserted central catheters; PAC: pulmonary artery catheters; PICC: peripherally inserted central catheters.

## Competing interests

The authors declare that they have no competing interests. The peripheral catheters were donated without restriction by the AngioDynamics Corporation.

## Authors' contributions

HEL participated in the design of the study, performed the study, helped with the statistical analysis, and drafted the manuscript. TTD conceived of the study, participated in the design of the study, and helped draft the manuscript. SQS participated in the design of the study, helped with the statistical analysis, and helped draft the manuscript. BLG participated in the design of the study. All authors read and approved the final manuscript.

## Pre-publication history

The pre-publication history for this paper can be accessed here:

http://www.biomedcentral.com/1471-2253/10/18/prepub
